# Patient-derived tissue slice cultures from endoscopic biopsies as a translational ex vivo model for inflammatory bowel diseases

**DOI:** 10.1007/s10238-026-02209-0

**Published:** 2026-06-22

**Authors:** Julia Werner, Laura Schiller, Claudia Müller, Jörg Lehmann, Jens Przybilla, Tobias Schlosser, Albrecht Hoffmeister, Sonja Kallendrusch, Cica Vissiennon

**Affiliations:** 1 Institute of Medical Physics and Biophysics, Faculty of Medicine, Leipzig University, Leipzig , Germany; 2 Repha GmbH Biologische Arzneimittel, Langenhagen, Germany; 3 Fraunhofer Institute for Cell Therapy and Immunology, Department of Preclinical Development and Validation, IZI, Leipzig, Germany; 4 Fraunhofer Cluster of Excellence Immune-Mediated Diseases CIMD, Leipzig, Germany; 5 Clinical Trial Centre Leipzig, Faculty of Medicine, Leipzig University, Leipzig, Germany; 6 Division of Gastroenterology, Medical Department II, University of Leipzig Medical Center, Leipzig, Germany; 7 Institute of Clinical Research and Systems Medicine, Health and Medical University, Potsdam, Germany

**Keywords:** Inflammatory bowel diseases, Patient-derived intestinal tissue slice cultures, Patient-stratified pharmacology, Ex vivo drug response

## Abstract

Effective inflammatory bowel disease (IBD) management increasingly relies on patient-stratified therapeutic approaches, yet clinically accessible platforms for ex vivo pharmacological evaluation remain limited. This study establishes patient-derived tissue slice cultures from routine endoscopic biopsies (ePDTC), as a potential translational platform for individualized drug response assessment. ePDTC were generated from routine colonic biopsies of ulcerative colitis (UC, *N* = 11), Crohn’s disease (CD, *N* = 7), and non-IBD control patients (*N* = 3). Tissue slices (350 μm high) were cultured at an air-liquid interface for 24 and 48 h, assessed for viability, immune cell composition, transcription factor activation, and mediator release profiles. Pharmacological responsiveness was evaluated using budesonide (1 µM) and myrrh extracts (50-100 µg/mL). Structural integrity was preserved in 88.4% of ePDTC at 24 h after start of cultivation with low apoptosis rates (median: 1.21%), but declined to 56.0% of tissues at 48 h. ePDTC preserved disease-specific inflammatory signatures, including distinct neutrophil patterns between UC and CD subtypes. Budesonide significantly reduced 17 of 24 inflammatory mediators after 24 h (adjusted *P* < 0.001) with indication of disease subtype-specific response patterns in principal component analysis. Myrrh extracts demonstrated concentration-dependent responses. Biopsy-derived ePDTC provide a clinically accessible ex vivo platform that preserves native tissue complexity and demonstrates the capacity to capture inter-individual pharmacodynamic heterogeneity within a 24-hour experimental window. This provides a biological basis for future patient-stratified pharmacological evaluation.

**Supplementary Information:**

The online version contains supplementary material available at 10.1007/s10238-026-02209-0.

## Introduction

Inflammatory bowel disease (IBD), comprising Crohn’s disease (CD) and ulcerative colitis (UC), is a chronic, multifactorial inflammatory disease with relapsing and clinically heterogeneous courses. Clinical management of IBD is challenged by substantial inter-individual variability in treatment response, including primary nonresponse and secondary loss of response, which vary by disease phenotype, prior therapy, and therapeutic class. Although the therapeutic landscape has expanded to include biologics, small molecules, and immunomodulators, response patterns remain highly heterogeneous across patients, drugs, and disease subtypes [1] underscoring the rationale for personalized therapeutic approaches [2].

However, reliable strategies for guiding therapy selection based on individual patient biology and for monitoring treatment response in routine practice remain limited, and current biomarker-based approaches have not yet translated into routine decision support. Consequently, there is active interest in developing functional platforms that can directly assess individual drug responses at the tissue level, with the goal of informing personalized treatment decisions in disease-relevant contexts [3]. Notably in oncology, functional precision medicine approaches using ex vivo drug testing on patient-derived tissue have demonstrated predictive value for clinical outcomes [4]. However, equivalent platforms for immune-mediated inflammatory diseases remain largely undeveloped.

Current functional models for patient-level drug testing in IBD have inherent limitations: patient-derived intestinal organoids preserve primary epithelium, but often lack resident immune cells and stromal interactions [5, 6]. Endoscopic biopsies are readily obtained, yet their viability in ex vivo cultivation is limited [7] and the constrained tissue volume necessitates single-treatment testing per biopsy, precluding direct comparative pharmacological evaluation within individual patients. Precision-cut intestinal slices (PCIS) generated from surgical resections maintain tissue architecture, viability, and resident immune populations, allowing for the direct evaluation of disease-specific cytokine patterns and testing of therapeutic agents directly in the inflamed tissue. However, current surgical PCIS models rely on large resection specimens, which are only available from patients with advanced, refractory disease undergoing surgery, thus limiting their routine accessibility [8, 9].

Beyond the challenges in therapeutic optimization, the evidence gap extends to complementary medicinal approaches. Many IBD patients utilize complementary therapies, particularly herbal products, motivated by concerns regarding the efficacy and adverse effect spectrum of conventional therapeutics [10, 11]. As a notable example, a randomized controlled trial demonstrated that an herbal combination of myrrh, coffee charcoal, and chamomile flower achieves clinical outcomes comparable to standard therapy in ulcerative colitis remission maintenance over a 12-month period [12]. Mechanistic studies in epithelial cell models demonstrate anti-inflammatory and barrier-stabilizing properties of myrrh extracts [13–15]. However, validation of these effects in complex multicellular human tissue preserving the native immune environment is lacking.

To address these translational gaps, this study introduces *patient-derived tissue slice cultures from endoscopic biopsies* (ePDTC), as a clinically accessible ex vivo platform for pharmacological evaluation in IBD. Specific objectives were (1) to develop and characterize a ePDTC system from routine endoscopic biopsies with preserved tissue architecture, immune cell populations, and inflammatory signaling capacity, and (2) to evaluate its capacity for detecting patient-specific drug response patterns using budesonide as a reference compound and myrrh extracts as a representative herbal medicinal compound requiring mechanistic validation in human tissue.

## Materials and methods

### Materials

Dulbecco’s Modified Eagle Medium: F12 Advanced (DMEM/F-12 Advanced), Dulbecco’s phosphate-buffered saline (DPBS), Fetal Bovine Serum (FBS), L-Glutamine, Penicillin-Streptomycin (PenStrep, 10.000 U/mL) and Roswell Park Memorial Institute 1640 Medium (RPMI1640) were purchased from Gibco (Life Technologies, Darmstadt, Germany). Budesonide, citric acid, dimethyl sulfoxide (DMSO), Hoechst 33342 and xylene were obtained from Sigma Aldrich (Taufkirchen, Germany). Paraffin, tri-sodium citrate dihydrate and Amphotericin B (AmphoB) were acquired from Carl Roth, Karlsruhe, Germany.

Primary antibodies to the following target proteins were used: CD68 (mouse; 1:500; DAKO, Santa Clara, California), neutrophil elastase (rabbit; 1:100; abcam, Cambridge, United Kingdom), STAT3 (mouse; 1:200; Cell Signaling Technology, Leiden, The Netherlands), phosphorylated STAT3 (rabbit; 1:200, Cell Signaling Technology), NFκB p65 (rabbit; 1:800; Cell Signaling Technology), phosphorylated NFκB p50 (Ser337; mouse; 1:200; Santa Cruz Biotechnology, Dallas, Texas), cleaved PARP (cPARP; rabbit; 1:100; abcam), and CD163 (rabbit; 1:200; abcam).

Secondary antibodies and detection systems included EnVision™ Detection System with Peroxidase/Diaminobenzidine (DAB) for rabbit/mouse (DAKO), goat-anti-mouse 568 and goat-anti-rabbit 647 (Alexa Fluor, Invitrogen, Life Technologies, Darmstadt, Germany), and normal goat serum (Biozol, Hamburg, Germany).

## Myrrh extract

Ethanolic extracts of myrrh (*Commiphora myrrha* Engl., Fam. Burseraceae) were prepared from the gum resin as previously described by Weber et al., 2020 [15]. Its chemical profile was comprehensively analyzed by Kuck et al. [16–18]. For the present experiments, dried extract was dissolved in DMSO and diluted to the indicated concentrations in culture media.

### Study design and ethics

This prospective experimental ex vivo study was approved by the Ethics Committee of the Medical Faculty, Leipzig University (reference numbers: 120/20-ek and AZ 370/13-ff). Patients meeting inclusion criteria were informed about the study at least 24 h before their scheduled endoscopy and provided written informed consent prior to study participation at the Clinic of Gastroenterology, University Hospital Leipzig. All participants were required to be at least 18 years of age. During routine endoscopic procedures, IBD patients provided additional tissue biopsies. The study involved minimal additional risk beyond routine endoscopic procedures. Patient data and tissue samples were transferred to the research team in pseudonymized form to protect patient identity.

### Tissue collection and processing

#### Patient specimens

Biopsies were obtained from patients with an established diagnosis of ulcerative colitis (N = 11) or Crohn’s disease (N = 7) during routine endoscopic procedures. Biopsies were taken from each section of the colon of all patients. Immediately following collection, biopsies were transferred into cooled culture medium (DMEM/F-12 Advanced supplemented with 15% FBS, 2 mM L-Glutamine, 100 U/mL penicillin, 100 µg/mL streptomycin, 250 ng/mL AmphoB) and transported on ice to minimize tissue degradation. Upon arrival in the laboratory, specimens were promptly washed in RPMI1640 medium containing PenStrep (100 U/mL penicillin, 100 µg/mL streptomycin) and AmphoB (250 ng/mL).

#### Control tissue

Control tissue samples were obtained from adjacent tissues (5 cm distance to tumor) of three patients receiving colon tumor resections through the Leipzig Medical Biobank, University of Leipzig Medical Center. Precise sections with a defined diameter of 3 mm were obtained using biopsy punches (pfm medical, Cologne, Germany) after slicing. The transfer and preparation procedures followed the same protocols established for patient biopsies to ensure consistency in handling and processing conditions.

#### Patient-derived tissue slice cultures from endoscopic biopsies (ePDTC): Preparation and culture

All tissue processing was performed in a laminar flow biosafety cabinet and with autoclaved instruments to maintain sterile conditions. Following washing procedures tissue specimens were sectioned into 350 μm thick slices using a McIlwain tissue chopper (Model TC752, Cavey Laborator Engineering Co. Ltd., Guildford, England). Individual slices were carefully separated under stereo microscopic guidance using sterile forceps and scalpel to ensure minimal mechanical damage.

Each tissue slice was placed on a sterile culture membrane insert at an air-liquid interface under standard culture conditions (24-well plates: 250 µL Millicell inserts, hydrophilic PTFE, 0.4 μm pore size; Merck, Darmstadt, Germany). Three to nine slices per IBD biopsy were cultured in 24-well plates for patient samples or 6-well plates for control tissue (TPP Techno Plastic Products, Trasadingen, Switzerland).

Treatment with budesonide (1 µM in 0.01% DMSO in culture medium) or myrrh extract (50 and 100 µg/mL in max. 0.1% DMSO) was added directly to the culture media for 24-48 h.

### Histological and immunological analyses

#### Tissue processing and histology

Following cultivation periods of 24–48 h, ePDTC were fixed overnight in 4% paraformaldehyde prepared by diluting methanol-free 16% formaldehyde solution (w/V; Pierce Biotechnology, Rockford, Illinois) in DPBS. In addition, ePDTC at t = 0 h were fixed to control for baseline characteristics.

Fixed ePDTC were processed through standard histological procedures, finally sectioned to 5 μm. Hematoxylin and eosin (HE) staining was performed to evaluate tissue orientation, overall tissue morphology and structural preservation.

#### Immunohistochemical analysis

Antigen retrieval was performed by heating sections in citrate buffer (pH 6.0). Immunohistochemical staining was conducted using the EnVision™ Detection System. After blocking, primary antibodies were diluted in DAKO real antibody diluent and applied for 60 min at room temperature. After three washes with wash buffer, sections were incubated with EnVision™ horseradish peroxidase-conjugated secondary antibody for 30 min at room temperature.

Visualization was achieved using DAKO DAB+ Chromogen diluted 1:50 in DAKO real substrate buffer, counterstained and mounted for microscopic analysis.

Digital slide scanning was performed using Pannoramic Scan and Viewer (3D histech, Budapest, Hungary). Quantitative analysis was conducted using QuPath software (v0.4.4, University of Edinburgh) with H-DAB image processing. Color deconvolution parameters were automatically estimated for each tissue section. Specific detection algorithms were applied: CD68-positive macrophages were quantified using “detect positive staining” function (downsample factor 1, hematoxylin threshold 0.22 OD units, DAB threshold 0.3 OD units), neutrophil granulocytes via “positive cell detection,” (hematoxylin OD, sigma 1, cell expansion 5 μm, score compartment: cytoplasm DAB OD max, threshold 0.43) in 3–5 regions of interest of the lamina propria. Transcription factors (STAT3, NFκB, and phosphorylated forms) were detected via “positive cell detection” (sum optical density or hematoxylin OD (for NFκB), sigma 1, cell expansion 5 μm, score compartment: nucleus DAB OD max, threshold 0.2–0.3 individual for each tissue) in the lamina propria and epithelium.

#### Immunofluorescence

For immunofluorescence analysis, deparaffinized sections were washed with 0.3% phosphate-buffered saline (PBS)/TritonX solution and blocked with normal goat serum for 30 min. Primary antibodies were diluted in 0.5% bovine serum albumin in PBS and incubated overnight at 4 °C. After washing secondary antibodies (goat-anti-mouse 568 and goat-anti-rabbit 647, Alexa Fluor series) were applied and nuclear counterstaining was performed by Hoechst 33,342.

Pictures were taken with an Olympus BX51 microscope. Three to five representative images per tissue slice were captured at 20x magnification. Quantitative analysis was performed using ImageJ with stain-specific segmentation algorithms [19], as described by Sönnichsen et al. [8]. Calculations included apoptotic cell fractions (cPARP-positive nuclei per total Hoechst-positive nuclei) and macrophage differentiation status (CD68^+^/CD163^+^ cells as fraction of total CD68^+^ population).

#### Mediator release analysis

Culture supernatants were collected after 24 h of cultivation, immediately centrifuged at 3,000 rpm for 5 min to remove cellular debris, and stored at -80 °C until analysis. Immune mediator quantification was performed using customized Luminex multiplex assays (R&D Systems, Minneapolis, Minnesota) according to manufacturer’s protocols.

Twenty-four analytes were measured to provide a broad coverage of pathophysiological processes central to IBD: pro-inflammatory cytokines driving Th1/Th17 and innate immune responses (TNFα, IFNγ, IL-1β, IL-2, IL-6, IL-17/IL-17 A, IL-23, IL-33, MIF); chemotactic cytokines mediating mucosal leukocyte recruitment (IL-8, CXCL5/ENA-78, CXCL9/MIG, MCP-1); growth and differentiation factors regulating granulopoiesis, macrophage differentiation, and stress responses (G-CSF, GM-CSF, GDF-15, Lipocalin-2); immunoregulatory cytokines representing anti-inflammatory and Th2 counter-responses (IL-4, IL-10); and modulatory or anti-inflammatory mediators involved in mucosal repair, epithelial integrity, and fibrinolytic regulation (TFF3, Angiogenin, VEGF, uPAR, Serpin-E1). Due to manufacturing constraints of the multiplex platform, the analytes were distributed across two separate, pre-configured assay panels optimized for sensitivity within their respective concentration ranges. Signal acquisition was performed on a Luminex FLEXMAP 3D instrument with all samples measured in duplicate.

Mediator concentrations (pg/mL) were determined using xPonent software (version 4.2, R&D Systems) based on standard curve analysis. Samples falling below or above standard curve ranges were excluded from analysis (75 out of 1224; 6.13%). To account for tissue size variations, the corresponding tissue area (µm²) was determined from HE-stained sections, and mediator release was normalized to tissue area for comparative analysis.

### Statistical analysis

Sample size (*N* = 18 IBD patients) was based on patient availability as well as ethical constraints of collecting additional biopsies during routine endoscopy and deemed appropriate for this feasibility study to establish proof-of-concept.

Statistical analyses were performed using R version 4.4.2 within the RStudio integrated development environment.

Linear mixed models (LMM) were calculated using the *lme4* R-package to account for the nested data structure. The nested data structure comprises the category patient, which contains subcategory biopsy, and at the third level the category of slices. Throughout this manuscript, *N* denotes the number of patients, *n* the total number of endoscopic biopsies examined, and *m* the number of individual slices analyzed. Data underwent logarithmic transformation to achieve normal distribution, verified through Shapiro-Wilk testing (α = 0.05). In the LMM framework, the individual detected target (antibody/mediator) was considered the dependent variable and the influence of the time, subgroup (IBD vs. control) or treatment was specified as a fixed effect variable. A nested random effect structure was specified to account for within-patient correlations among related samples from the same biopsy, thereby controlling for patient-specific variations (see *Online Resource 1*). To account for multiple testing across the 24 mediators when investigating the pharmacological effect of budesonide, p-values obtained from individual linear mixed models were adjusted using the Benjamini-Hochberg procedure to control the false discovery rate (FDR). This approach limits the expected proportion of false positives among the set of statistically significant results. Adjusted *P*-values (*q*-values) below 0.05 were considered statistically significant [20].

GraphPad PRISM (Version 10.6.0 for Windows; GraphPad Software, Boston, Massachusetts) was used to represent values from different biopsies of one patient, and consequently averaged. Median as well as the interquartile range (lower to upper limit: Q_1_-Q_3_) was reported to account for heterogeneity between individuals. Due to the paired nature of measurements (baseline at 0 h vs. treatment at 24 h within individual biopsies), baseline correction was applied by calculating fold-change ratios (24 h/0 h values) for all quantitative outcomes. This transformation normalized baseline values to 1.0, enabling direct comparison of treatment effects across patients.

Additional within-cohort statistical analyses for the IBD group were performed using the *dplyr* R-package to facilitate data transformation and principal component analysis (PCA) calculations. Prior to PCA, missing values were imputed using group-wise medians: data was split by treatment group, and missing numeric values were replaced with the median of each variable within that group, preserving group-specific distributions and minimizing cross-group bias. Data was logarithmically transformed, mean values were calculated for individual patients, and datasets were scaled appropriately. The *ggplot2* R-package was used for visualization.

## Results

### Patient characteristics and ePDTC generation

**Fig. 1:**
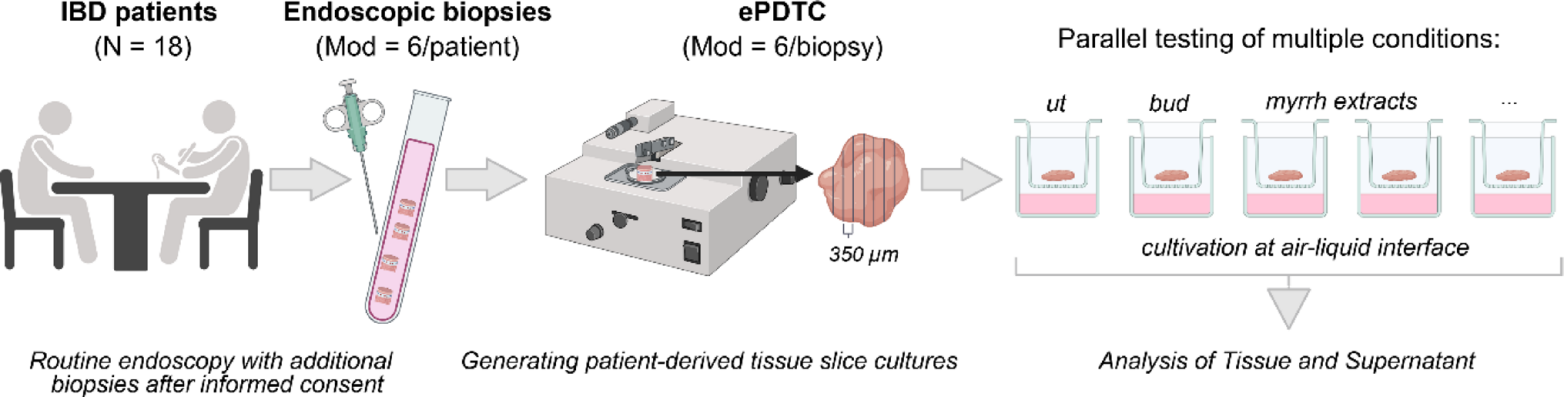
Schematic workflow from clinical biopsy to ex vivo cultivation. After providing informed consent, patients undergo a routine endoscopy during which the gastroenterologist takes additional biopsies. Each endoscopic biopsy is cut into defined intestinal tissue slices (350 μm), which are individually placed on membrane inserts and cultured under standardized conditions. This setup allows multiple treatment conditions to be tested in parallel on slices derived from a single biopsy. After cultivation, tissue and supernatant can be analyzed. (ut: untreated sample; bud: budesonide treated sample; created in BioRender. Kosel, L. (2026): https://BioRender.com/k691vrd)

Biopsies were obtained from 18 IBD patients comprising 11 patients with ulcerative colitis (61.1%) and 7 with Crohn’s disease (38.9%) and processed as outlined in Fig 1. The cohort had a median age of 40.5 years (range: 28–68 years) with equal gender distribution (50% female, 50% male) (Table 1). Most patients (72.2%) were in mild-moderate inflammatory episodes or clinical remission at the time of biopsy collection. Current medication regimens varied across the cohort, with 44.4% receiving biologicals and immunosuppressants, 55.5% on glucocorticoids and anti-inflammatory drugs, and 11.1% using probiotics as complementary approaches. Detailed medication profiles for each patient are provided in *Supplementary Information* (Online Resource 2).

**Table 1:** Patients specimens. Disease activity: H: High, M: Mild-moderate, R: Remission and ca.: colorectal cancer (tissue from control patients); Bud: Budesonide 1 µM; MY: treatment with myrrh-extract (50 and 100 µg/mL); IF: Immunofluorescent staining; IHC-IC: Immunohistochemistry of immune cells; IHC-IP: Immunohistochemistry of inflammatory pathways

No				Ex vivo cultivation		Investigation methods				
	Age, Sex	Disease	Activity	Time [h]	Treatment	Tissue integrity	IF	IHC-IC	IHC-IP	Multiplex
**1**	28, m	UC	H	24, 48		x				
**2**	37, f	UC	H	24, 48		x				
**3**	41, m	UC	M	24, 48		x				
**4**	47, m	UC	R	24, 48		x				
**5**	59, m	UC	M	24, 48		x				
**6**	31, m	CD	R	24, 48		x				
**7**	29, m	CD	H	24, 48	Bud	x	x	x	x	x
**8**	40, m	CD	R	24, 48	Bud	x	x	x	x	x
**9**	63, f	UC	M	24, 48	Bud	x	x	x	x	x
**10**	44, f	CD	R	24, 48	Bud	x	x	x	x	x
**11**	37, f	UC	R	24, 48	Bud	x	x	x	x	x
**12**	31, f	UC	H	24, 48	Bud	x	x	x	x	x
**13**	47, f	CD	M	24	Bud, MY		x		x	x
**14**	66, f	CD	R	24	Bud, MY		x		x	x
**15**	40, f	UC	R	24	Bud, MY		x		x	x
**16**	38, f	CD	H	24	Bud, MY		x		x	x
**17**	59, m	UC	M	24	Bud, MY		x		x	x
**18**	47, m	UC	M	24	Bud, MY		x		x	x
**C1**	63, m	ca.	-	24, 48	Bud, MY			x		
**C2**	84, f	ca.	-	24, 48	Bud, MY			x		
**C3**	51, f	ca.	-	24, 48	Bud, MY			x		

A total of 96 biopsies (3–10 per patient, mode at 6) were collected and processed using a tissue chopper within approximately 2.5 h (149.9 min ± 37.5 min) to generate 350 μm thick slices. This yielded 582 ePDTC (1–13 per biopsy, mode at 6). Following cultivation and fixation procedures, 534 samples (92.8% recovery rate) were successfully embedded in paraffin for subsequent histological analyses. Control tissue from three patients without diagnosed inflammatory disorders was processed similarly (#C1-C3).

### Tissue integrity and viability

**Fig. 2:**
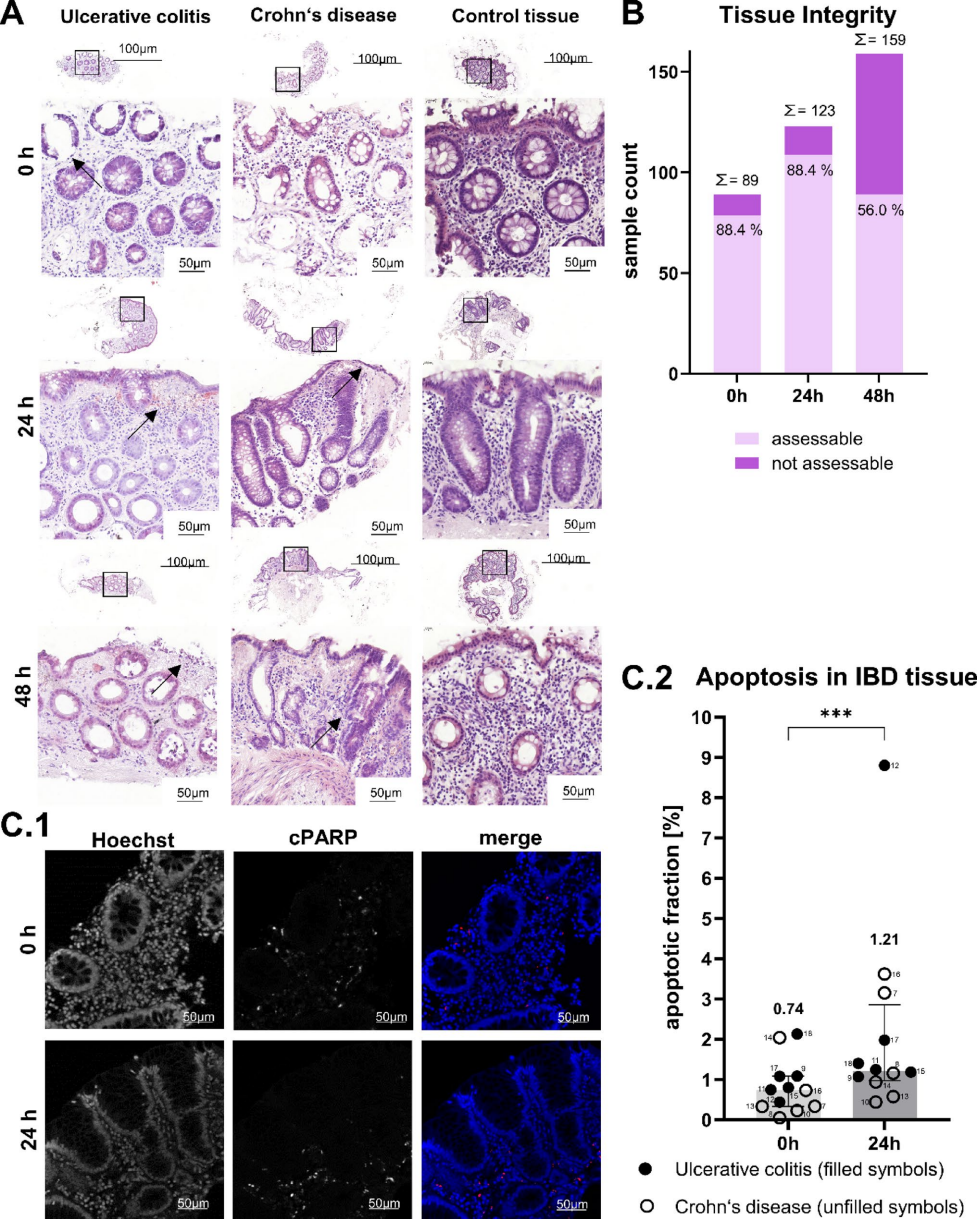
Cultivation of ePDTC from IBD biopsies. (**A**) HE staining of untreated patient-derived intestinal slices (ePDTC) from ulcerative colitis (UC), Crohn’s disease (CD), and control tissue at 0, 24, and 48 h. Arrows indicate erosions, ulcerations, and epithelial flattening (scale bar = 100 μm; 20x magnification). (**B)** Proportion of assessable ePDTC from *N* = 12 IBD patients (#1–12) with *n* = 61 biopsies and m = 243 slice cultures analyzed (0 h: m = 97; 24 h: m = 65; 48 h: m = 81). (**C)** Apoptotic fraction in *N* = 12 IBD patients (#7–18) with *n* = 45 biopsies and m = 85 slice cultures analyzed (0 h: m = 45; 24 h: m = 40) determined by cleaved PARP immunofluorescence (red) with Hoechst 33,342 nuclear counterstain (blue). C.1: representative pictures (scale bar = 50 μm; 40x). C.2: Quantification of apoptotic nuclei before and after 24 h (median ± IQR). *Significance tested using* linear mixed models of log-transformed raw data (*** *P* < 0.001)

HE staining demonstrated well-preserved tissue architecture in ePDTC from UC, CD, and control samples at 0 and 24 h, with characteristic IBD-associated alterations (e.g., erosions, ulcerations, epithelial flattening) evident in diseased tissue (Fig. 2A 1A). After 24 h, 88.4% of IBD samples maintained structural integrity comparable to baseline (Fig. 2B 1B). Tissue integrity declined significantly after 48 h cultivation time, establishing 24 h as the optimal cultivation window for subsequent analyses. Tissue viability was assessed using cleaved PARP immunofluorescence as a marker of apoptotic cell death. A marginal increase was observed in apoptosis level from 0.74% (median; Q_1_-Q_3_: 0.33–1.09%) in freshly collected tissue (0 h), increasing to 1.21% (0.97–2.86%) after 24 h of cultivation (*N* = 12 patients, #7–18; *n* = 45 biopsies; m = 85 slice cultures; LMM: *P <* 0.001). Three patients with high disease activity (#07: 3.15%, #12: 8.80%, #16: 3.62%) demonstrated apoptosis rates above the cohort median.

### Immunological characterization

#### Immune cell infiltration

**Fig. 3:**
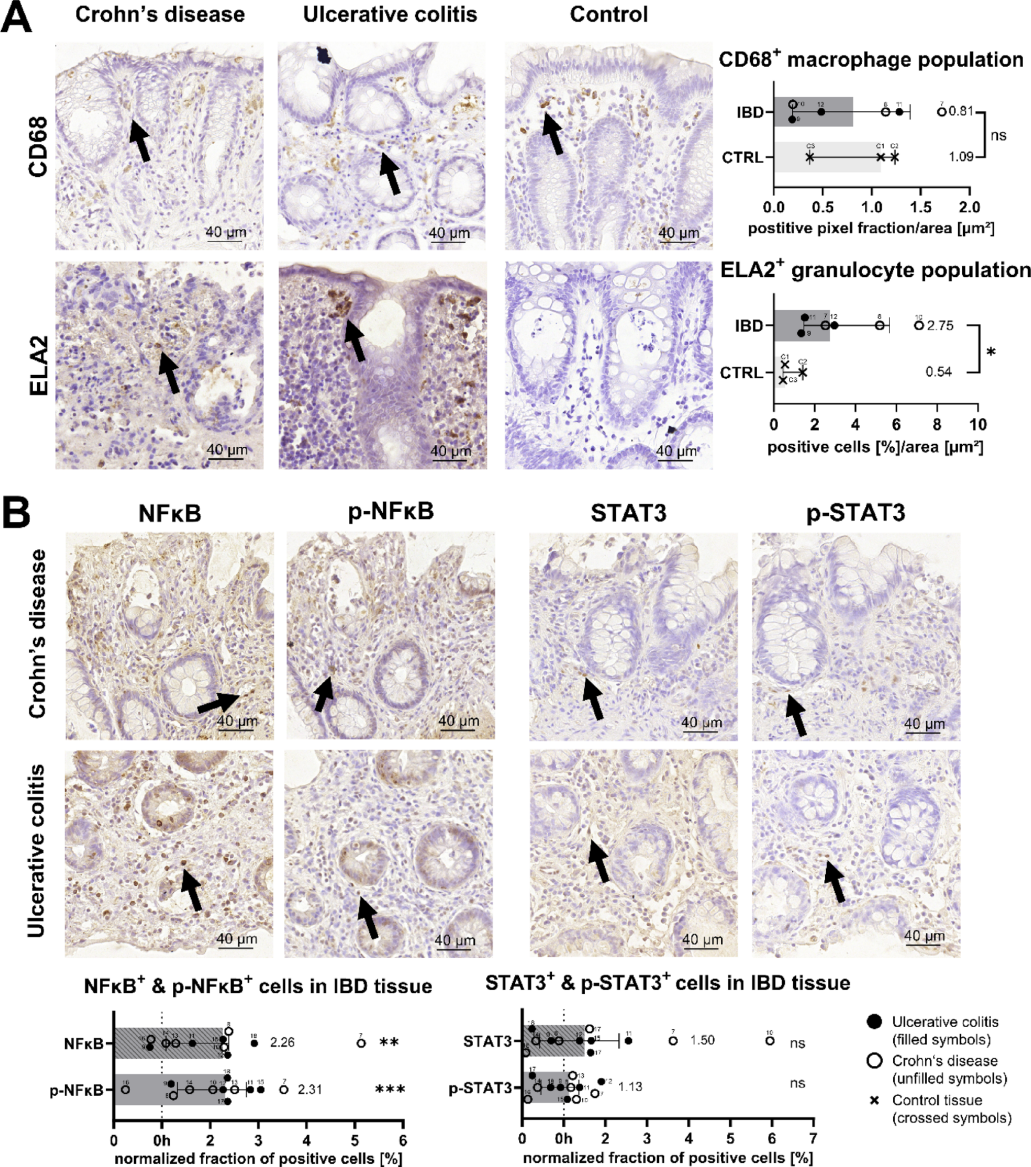
Immunohistochemical analysis of immune cells and transcription factors.IHC staining of different targets by DAB (brown) after 24 h cultivation, respectively. Nuclei are counterstained with hematoxylin (blue). Positive cell fractions after 24 h were detected using QuPath. (**A**) CD68-positive macrophage density (positive pixel area [%]) and neutrophil elastase-positive granulocyte fraction (positive cell [%]) in *N* = 6 IBD patients (#7–12; *n* = 13 biopsies and m = 13 slice cultures) vs. control tissue (*N* = 3; *n* = 3 samples and m = 9 slice cultures) ePDTC after 24 h (median ± IQR). (**B)** Transcription Factor activation: NFκB, phosphorylated NFκB (p-NFκB), STAT3 and phosphorylated STAT3 (p-STAT3) for *N* = 12 IBD patients (#7–18) (except NFκB: *N* = 11) with *n* = 30 biopsies and m = 54–56 slice cultures analyzed (0 h: m = 30; 24 h: m = 24–26). Fraction of positive cells in NFκB and STAT3 activation after 24 h normalized to 0 h (marked at x = 1); median ± IQR. Comparison between cohorts (A: IBD vs. CTRL) or intra-cohort (B: 0 h vs. 24 h) significance tested using linear mixed models of log-transformed data at raw data level (*** *P* < 0.001, ** *P* < 0.01, * *P* < 0.05, *P* ≥ 0.05 = not significant, ns)

CD68-positive macrophages were detected at similar densities in IBD tissue (*N* = 6; 0.81%, 0.19–1.39%) and control tissue (*N* = 3; 1.09%, 0.37–1.24%, LMM: *P* = 0.95) after 24 h of cultivation. Neutrophil elastase-positive granulocytes demonstrated significantly higher amount in IBD tissue (2.75%, 1.47–5.67%) versus non-inflamed control tissue (0.54, 0.44–1.42%; LMM: *P <* 0.05). At the individual level, UC patients in remission (09, 11) tended to show lower neutrophil counts, while CD patients in remission (08, 10) tended to show higher values (Fig. 3A 2A).

#### Transcription factor activation

Ex vivo cultivation induced significant NFκB pathway activation in IBD ePDTC (N = 12), with phosphorylated NFκB-positive cells increasing 2.31-fold after 24 h compared to baseline (Q1-Q3: 1.32–2.75, LMM: P < 0.001). Total NFκB expression also rose significantly (2.26-fold, 1.08–2.38, LMM: P < 0.01), while STAT3 activation remained unchanged (1.50-fold, 0.42–2.33, LMM: P = 0.77; Fig. 3B 2B).

#### Mediator release profiles

**Fig. 4:**
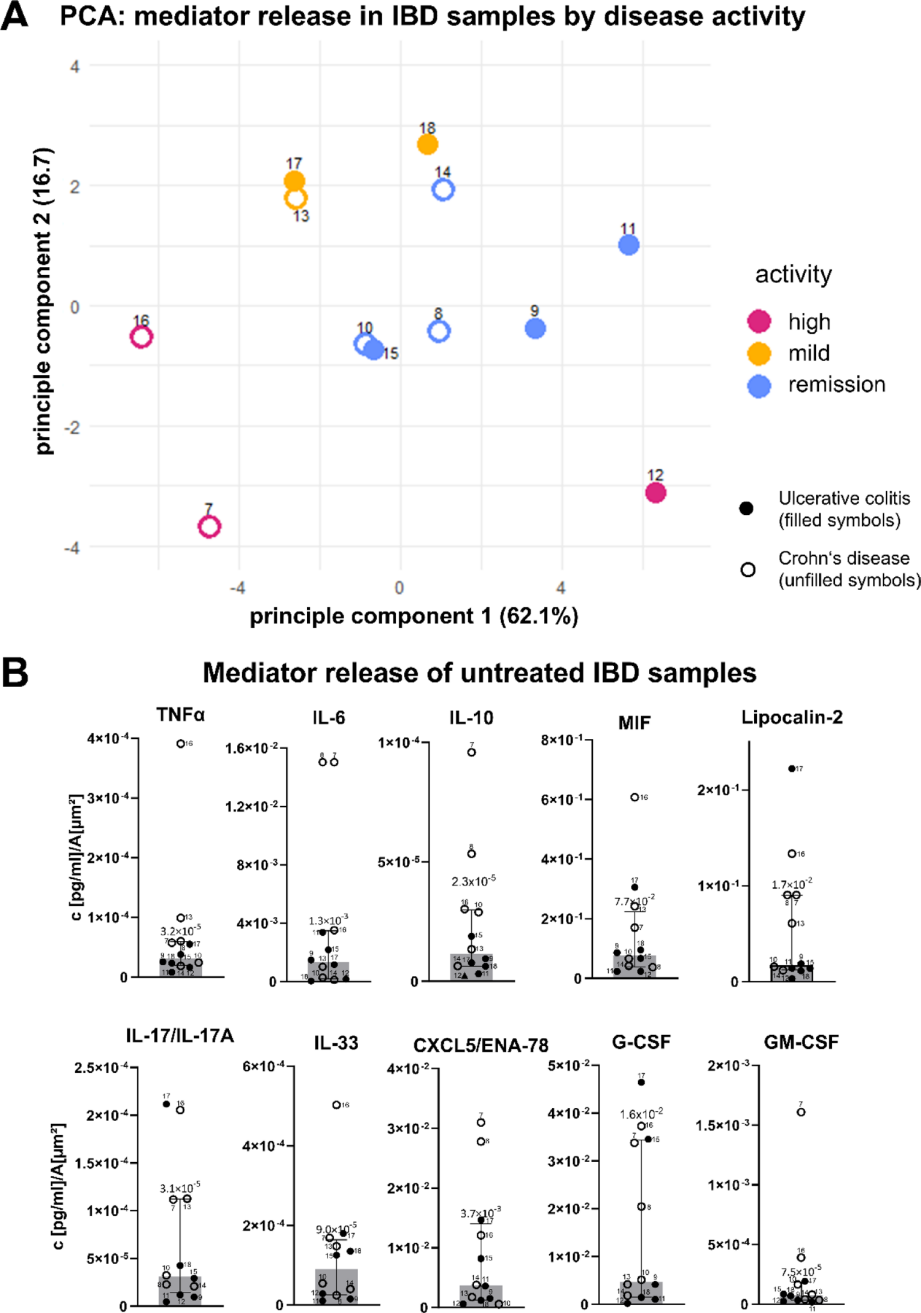
Immune mediator release of untreated IBD ePDTC samples. (**A**) Principal component analysis of 24 immune mediators, clustered by disease activity. **B.** Released concentration in [pg/mL] per area [µm²] of selected mediators (TNFα, IL-6, MIF, IL-17/IL-17 A, CXCL5/ENA78, IL-10, G-CSF, GM-CSF, Lipocalin-2) from ePDTC after 24 h cultivation. Data represent *N* = 12 IBD patients (#7–18) with *n* = 52 biopsies and m = 104 slice cultures analyzed. Median ± IQR. Excluded: 6.13% (75/1224) of measurements falling outside standard curve ranges. Full data set in *Supplementary Information (Online Resource 3)*

Multiplex protein analysis quantified 24 immune mediators in culture supernatants from slice cultures derived from 12 IBD patients (*n* = 51 biopsies) after 24 h of cultivation without treatment. Of 1224 total measurements 75 (6.13%) were excluded due to concentrations falling outside standard curve ranges. Principal component analysis of the complete mediator profile demonstrated clustering by disease activity, with high-activity patients forming distinct subgroups regardless of disease subtype. Patients #7 and #16 (CD, high activity) showed a significantly stronger expression on the first principal component (PC1 ≤ -4), while patient #12 (UC, high activity) showed a pronounced influence from the first principal component to the opposite direction (PC1 > 4; Fig. 4A 3A).

Normalized mediator concentrations spanned a broad dynamic range. Cytokines including TNFα, IL-10, and IL-17/IL-17 A were detected at lower concentration ranges (10^− 5^ pg/mL/µm²), while chemokines and growth factors including MIF, Lipocalin-2, and G-CSF were measured at higher concentrations (> 10^− 2^ pg/mL/µm²). Patients with active Crohn’s disease (#7, #16) consistently demonstrated the highest mediator concentrations across multiple analytes (Fig. 4B 3B).

### Pharmacological modulation

#### Budesonide

**Fig. 5:**
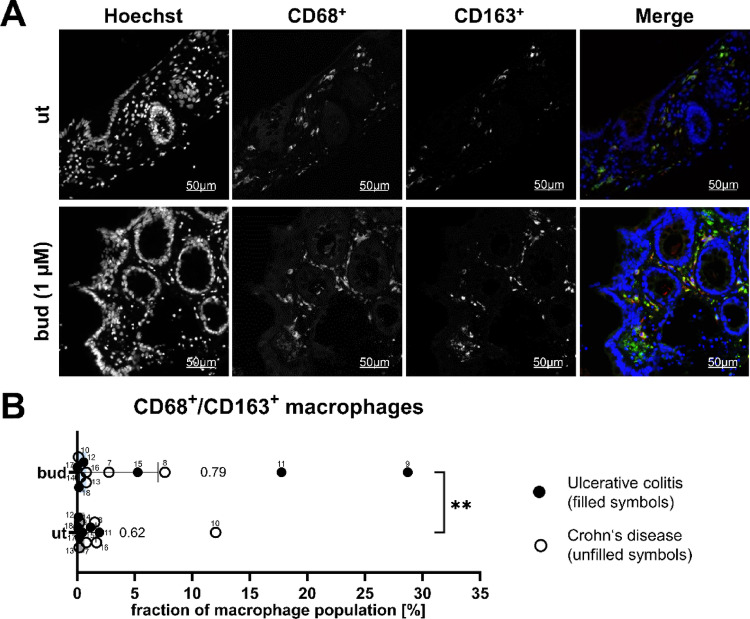
Macrophage polarization in IBD tissue after budesonide treatment. (**A**) Immunofluorescent staining with antibodies against CD68 as a pan-macrophage marker (green) and CD163 as a marker for differentiating macrophages (red), and Hoechst 33,342 (nuclei, blue). Differentiating macrophages are determined as nuclei with co-localization of CD68 and CD163 (scale bar = 50 μm; 20x). (**B**) Fraction of CD68^+^/CD163^+^ macrophages of total CD68^+^ population in untreated (ut) vs. budesonide (bud, 1µM)-treated conditions after 24 h. Data represent *N* = 12 IBD patients (#7–18) with *n* = 43 biopsies and m = 68 slice cultures analyzed (ut: m = 41; bud: m = 27). Median ± IQR. Significance tested using linear mixed models of log-transformed raw data (** *P <* 0.01)

Treatment of slice cultures with budesonide (1 µM) for 24 h preserved tissue integrity and maintained apoptosis rates comparable to untreated controls (median: 1.21% vs. 1.91%, respectively; data not shown). Hematoxylin and eosin staining revealed no adverse effects on tissue architecture. Macrophage polarization analysis using CD68/CD163 co-immunofluorescence showed significant differences (LMM: *P* < 0.01) between budesonide-treated samples (0.79% CD68⁺/CD163⁺ cells, Q_1_-Q_3_: 0.19-–7.03%) and untreated controls (0.62%, 0.19–1.65%). Individual patient responses varied, with three patients (#08, #09, #11) exhibiting above-median polarization values following budesonide treatment (Fig. 5,4).

**Fig. 6:**
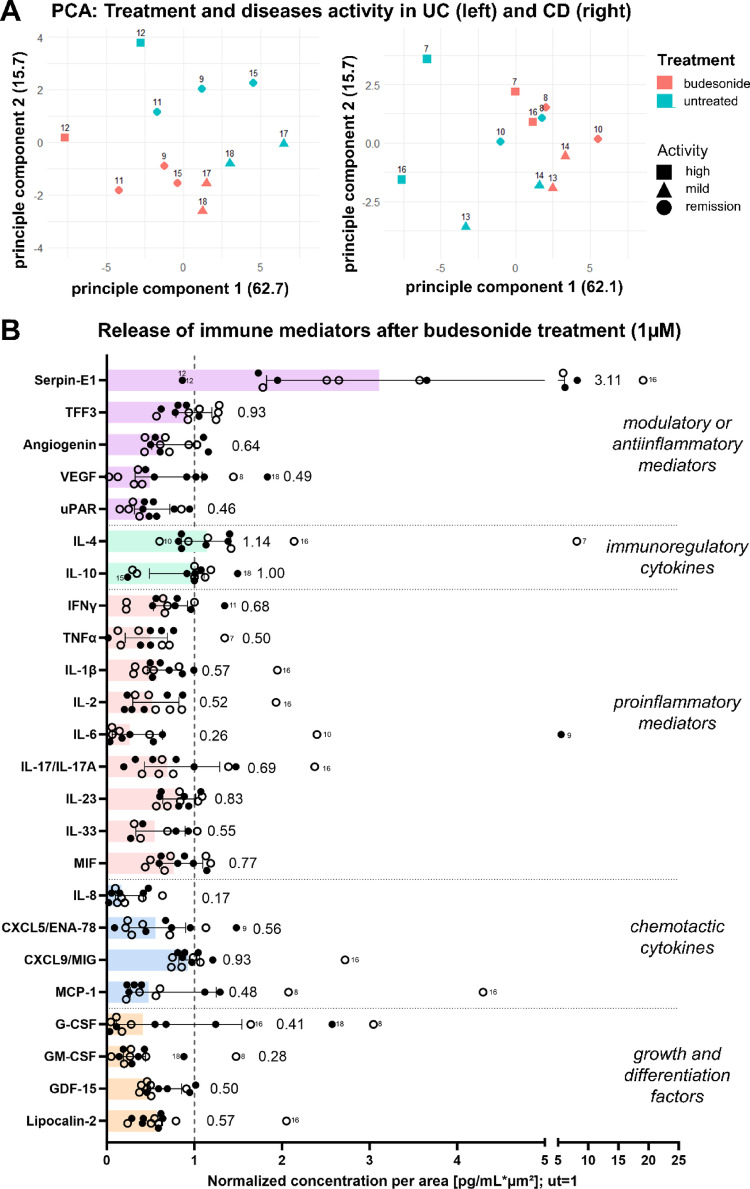
Mediator release of IBD tissue after budesonide treatment. (**A**) Principal component analysis of mediator profiles in UC (left) and CD (right) ePDTC following budesonide (1 µM, 24 h) or control treatment for *N* = 6 UC patients (#9, 11, 12, 15, 17, 18; *n* = 28 biopsies) and *N* = 6 CD patients (#7, 8, 10, 13, 14, 16; *n* = 24 biopsies). Symbol colors indicate disease activity levels (high, moderate, remission). **(B)** Fractional change in mediator release of 24 analytes after budesonide (1 µM, 24 h) treatment (normalized to untreated samples, ut = 1). Data represent *N* = 12 IBD patients (#7–18; *except IL-8: *N* = 11) with *n* = 52 biopsies and m = 85 slice cultures analyzed (ut: m = 49; bud: m = 36). *Median ± IQR*

Principal component analysis of mediator release profiles (*N* = 12 IBD patients; *n* = 52 biopsies; m = 104 slice cultures) suggests that ePDTC from UC patients exhibit more concordant mediator profile shifts following budesonide treatment, regardless of baseline disease activity. In comparison CD specimen showed greater inter-individual variability with active disease (high and moderate activity) shifting toward remission-like profiles, while ePDTC from patients in remission showed minimal changes (Fig 6A. 5A).

Quantitative analysis using linear mixed models with patient as random effect (*N* = 12 IBD patients; *n* = 52 biopsies; m = 85 slice cultures) and FDR-adjusted *P*-values (Benjamini-Hochberg method, *q* < 0.05) revealed highly significant reductions (*q* < 0.001) in 17 of 24 analytes, including key pro-inflammatory mediators (TNFα, IL-6, IL-8, IFNγ), chemotactic factors (CXCL5/ENA-78), growth factors (G-CSF, GM-CSF), and the anti-inflammatory cytokine IL-10. Additional mediators showing significant reductions included MIF, IL-1β, IL-17/IL-17 A, and IL-23 (*q* < 0.01), and TFF3 (*q* < 0.05). Serpin-E1 was the only mediator to show a significant increase following budesonide treatment (*q* < 0.001). IL-4 and CXCL9/MIG showed no significant response (*q* ≥ 0.05).

Pronounced inter-individual variability in budesonide response was observed. Certain patients exhibited marked mediator suppression: ePDTC from patient 16 (Crohn’s disease, high activity) for example, showed a reduction in key mediators (like TNFα: 0.16-fold), while conversely demonstrating increased release of other proinflammatory mediators (IL-2, IL-33, IL-1β, IL-17/IL-17 A) and growth factors (except GM-CSF). Also, tissue from patient 08 (Crohn’s disease, remission), showed paradoxical increases in key cytokines (IL-6, G-CSF, TNFα, MCP-1, and IL-1β; Fig. 6B 5B).

#### Myrrh extracts

To explore the model’s capacity to accommodate pharmacological testing of complex herbal extracts, slice cultures of six IBD patients (#13–18; *n* = 28 biopsies, m = 59 slice cultures) were treated with myrrh extracts at concentrations of 50 and 100 µg/mL for 24 h. Tissue integrity and apoptotic response were chosen as primary readouts, as these parameters allow assessment of tolerability and provide a pharmacologically interpretable signal in the context of previously published concentration-dependent cytotoxic effects of myrrh extract in intestinal epithelial cell models [13–15].

**Fig. 7:**
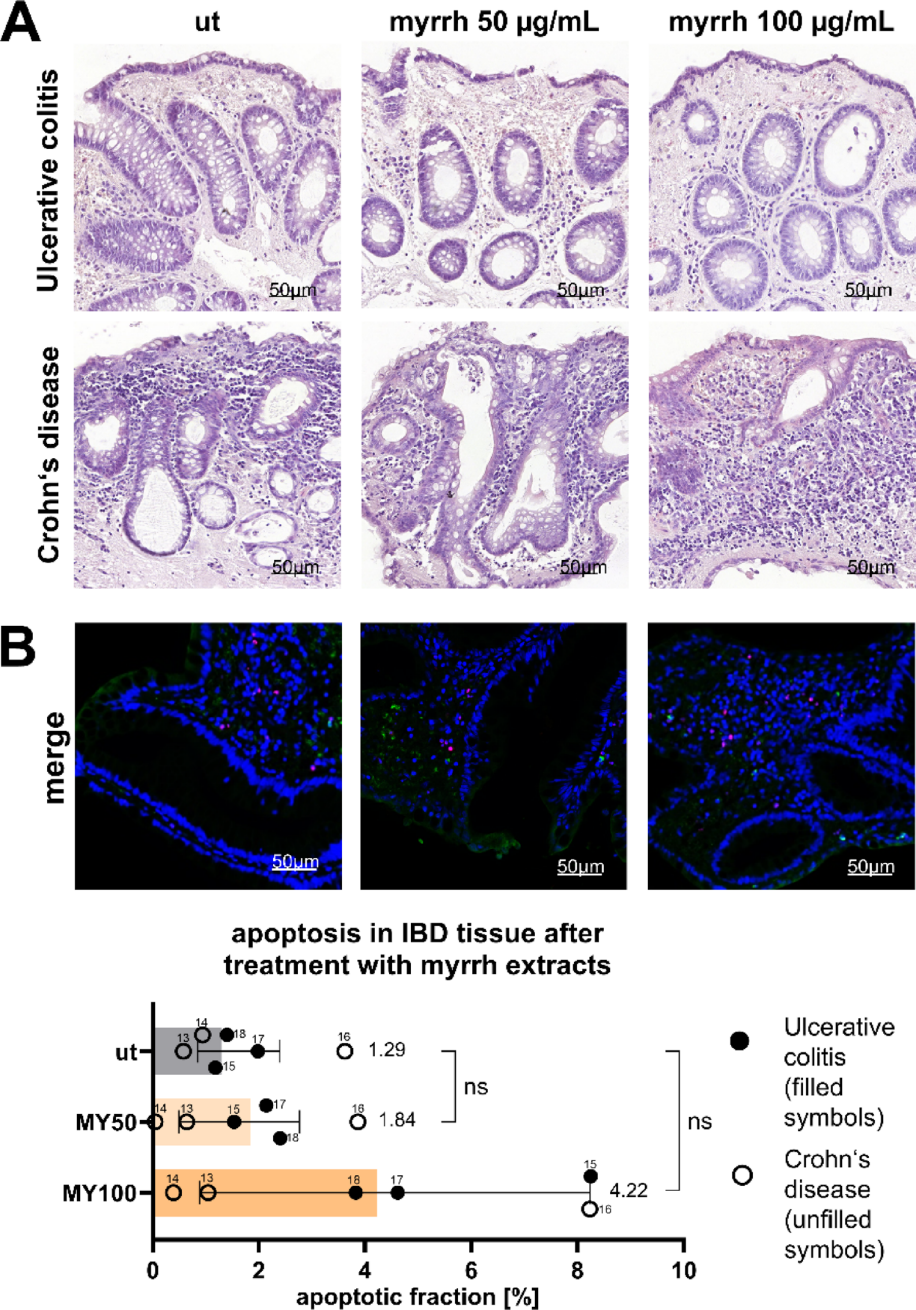
Effects of myrrh extracts on tissue integrity and viability of ePDTC from IBD biopsies. (**A**) HE staining of ePDTC after 24 h cultivation without (ut) or with myrrh extract (50 µg/mL, MY50; 100 µg/mL, MY100) (scale bar = 50 μm; 20x). (**B.1**) Cleaved PARP immunofluorescence (red) with Hoechst 33,342 (blue) showing apoptosis in ePDTC under each condition (scale bar = 50 μm; 20x). (**B.2**) Quantification of apoptotic nuclei after 24 h cultivation time of untreated samples (ut) or after treatment with 50–100 µg/mL myrrh extract (MY50, MY100). Data represent *N* = 6 IBD patients (#13–18) with *n* = 28 biopsies and m = 59 slice cultures analyzed (ut: m = 27; MY50: m = 15; MY100: m = 17). Median ± IQR. Significance tested using linear mixed models of log-transformed raw data (*P* ≥ 0.05 = not significant, ns)

Histological evaluation revealed no obvious effects on tissue preservation at either concentration compared to untreated controls. Both UC and CD samples maintained structural integrity, with preserved epithelial architecture and lamina propria organization comparable to baseline conditions (Fig. 7A 6A). However, treatment with 50 µg/mL myrrh extract resulted in apoptosis rates comparable to untreated controls (1.84%, 0.49–2.77% vs. 1.29%, 0.85–2.39%, respectively; LMM: *P* ≥ 0.05), the high concentration of 100 µg/mL showed an apoptotic cell fraction of 4.22% (0.88–8.24, LMM: *P* ≥ 0.05). Solvent control (DMSO 0.1%) showed no influence on tissue viability after 24 h compared to untreated samples (1.10%, 0.79–1.59%, LMM: *P* ≥ 0.05, data not shown).

Individual patient variability was notable across all treatment conditions. Patient #16 (Crohn’s disease, high activity, receiving ozanimod) consistently exhibited the highest cleaved PARP expression in untreated (3.62%), 50 µg/mL (3.87%), and 100 µg/mL (8.23%) conditions. The remaining patients showed variable responses to both concentrations, with some individuals demonstrating minimal changes while others exhibited moderate increases in apoptotic cell fraction at the higher concentration (Fig. 7B 6,7).

## Discussion

This feasibility study is the first to establish a *patient-derived tissue slice culture model* from routine endoscopic biopsies (ePDTC) of IBD patients, demonstrating preserved tissue architecture, functional immune cell populations, and robust pharmacological responsiveness over 24 h. By combining the accessibility of endoscopic sampling with the multicellular complexity required for meaningful pharmacological studies, this platform constitutes a proof-of-concept framework for capturing inter-individual pharmacodynamic heterogeneity in IBD tissue with the potential for future pharmacological studies of e.g. complementary drugs taken by patients or novel compounds requiring evidence-based validation in a human immune-competent setting.

Consistent with a previously published study we showed a threshold of ≤ 5% apoptosis after 24 h in ex vivo human colon cultures with the here established ePDTC method [21]. While surgical precision-cut tissue slices from gastric and esophagogastric cancer tissue can maintain viability for up to 6 days [22] Grieger et al. confirmed a comparable 24-hour viability window in precision-cut tissue slices derived from ileal resections of Crohn’s disease patients [9]. Yet, these approaches require extensive surgical procedures limiting accessibility. Organoid cultures, though expandable for weeks, lack resident immune cells, organotypic tissue architecture and stromal interactions limiting their utility for studying immune-epithelial interactions [23]. Whole biopsy explant methods, while valuable for certain applications, yield insufficient tissue for within-sample comparative testing, precluding detailed pharmacological assessment [7]. Our ePDTC model bridges these limitations by enabling parallel testing of multiple conditions (approx. 6 slices per biopsy) while preserving native tissue complexity through minimally invasive tissue collection.

ePDTC successfully recapitulated key IBD features while revealing clinically relevant patterns. Immunohistochemical analysis demonstrated stable CD68⁺ macrophage densities in both IBD and control tissue and a significant trend toward increased neutrophil infiltration in IBD samples. Notably, UC patients in remission (#09, #11) demonstrated the lowest neutrophil counts among IBD samples, while CD patients in remission (#08, #10) exhibited the highest values. This persistent neutrophil presence confirms reports that Crohn’s disease patients in clinical remission exhibit ongoing mucosal neutrophil infiltration [24], whereas ulcerative colitis remission typically correlates with neutrophil absence in histological indices [25].

The observed NFκB activation following ex vivo cultivation is consistent with a stress response induced by mechanical tissue processing and cultivation conditions, reflecting the functional integrity of inflammatory signaling pathways in our ePDTC model. The two-fold increase in phosphorylated NFκB-positive cells aligns with established mechanotransduction responses where mechanical stress activates NFκB through focal adhesion kinase-dependent mechanisms [26] and mirrors the mechanosensitive NFκB responses observed in murine cell models [27]. This pathway-specific reactivity confirms that our IBD ePDTC retain the capacity for dynamic transcription factor regulation. However, this induced NFκB baseline activation should be considered a confounding factor when interpreting downstream mediator release and drug effects. Accordingly, all pharmacological comparisons were made against matched untreated controls under identical conditions to account for this baseline activation. In contrast, STAT3 activation was not observed within our 24-hour culture window, likely reflecting the requirement for sustained IL-22 signaling that typically develops over days during inflammation, as demonstrated in both mouse colitis models and human intestinal tissue cultures [28].

Multiplex profiling of 24 immune mediators in ePDTC supernatants, normalized to tissue area, demonstrated clustering by disease activity in principal component analysis, with cytokines (TNFα, IL-17/IL-17 A, IL-10) detected at 10^− 5^ pg/mL/µm² and growth factors (MIF, G-CSF) exceeding 10^− 2^ pg/mL/µm^2^. This relative concentration pattern matches the cytokine hierarchy reported in ex vivo biopsy cultures [7], confirming the model’s fidelity in recapitulating the complex secretome of the intestinal mucosa.

Pharmacological validation with 1 µM budesonide preserved ePDTC viability and elicited significant reductions in 17 of 24 mediators, including TNFα, IL-6, IL-8, and IFNγ in line with glucocorticoid pharmacodynamics observed in human intestinal tissue [29], and organoid cultures [30]. This suppression profile recapitulates the canonical transrepression activity of the ligand-activated glucocorticoid receptor (GR) via NF-κB and AP-1 inhibition [31]. The concomitant increase in Serpin-E1 (PAI-1) release confirms GR-mediated transactivation through glucocorticoid response elements in the SERPINE1 promoter [32]. In addition, the significant rise in CD163⁺ macrophage polarization is consistent with GR-mediated macrophage reprogramming described in tissue repair contexts [33, 34].

Budesonide’s broad anti-inflammatory profile in our model mirrors clinical effects, inducing endoscopic remission in 46–84% of patients with active distal ulcerative colitis and clinical remission in 42–67% of patients with active Crohn’s disease [35]. Principal component analysis revealed clustering by disease activity and treatment condition, with UC ePDTC exhibiting more concordant mediator profile shifts compared to CD slices, which displayed greater inter-individual variability. Notably, individual ePDTC responses to budesonide varied considerably, with some patients (e.g., #16) exhibiting robust anti-inflammatory responses and others (e.g., #08, #09) displaying minimal or even paradoxical mediator changes. These inter-individual response patterns may partly reflect differences in concomitant medication profiles (Online Resource 2, *Supplementary Information*). For example, patient #16 was receiving ozanimod (sphingosine-1-phosphate receptor modulator) at the time of tissue collection, while patient #08 was on combination therapy with adalimumab 40 mg s.c. biweekly plus oral budesonide 3 mg/d. Although tissue was rinsed during processing, cellular drug effects from in vivo medication persist and can substantially modulate ex vivo responses. Biologics such as anti-TNF agents have been shown to alter glucocorticoid sensitivity in intestinal tissue [36], and chronic pre-exposure to oral budesonide (as in patient #08) may induce partial glucocorticoid receptor desensitization [37]. Thus, the observed variability likely reflects complex interactions between baseline disease activity, chronic medication effects, and acute ex vivo drug exposure. However, systematic attribution of ex vivo drug responses to specific medication classes was beyond the scope of this feasibility study and will require larger, stratified cohorts with balanced medication subgroup.

Myrrh extract was tested as a proof-of-concept application to explore the model’s suitability for evaluating pharmacological properties of complex herbal preparations, often taken by patients without knowledge of healthcare practitioners. The observed concentration-dependent increase in apoptotic cell fraction with no significant apoptosis increase at 50 µg/mL and only a modest rise at 100 µg/mL is consistent with cytotoxic thresholds previously identified in (intestinal epithelial) cell cultures [13–15, 38]. While these ex vivo concentrations exceeded those typically achieved systemically (bioavailability of myrrh constituents in gut tissue remains poorly defined), they establish a concentration range suitable for investigating direct mucosal effects. This addresses a critical need in complementary IBD medicine, where evidence-based evaluation of herbal therapeutics remains limited. A comprehensive characterization of the pharmacological effects of myrrh and the full herbal combination (myrrh, chamomile flower, coffee charcoal) in the ePDTC model is however subject of ongoing work and will be reported separately.

Our ePDTC model has several inherent constraints that must be acknowledged. As a feasibility study, ethical constraints limited patient enrollment and sample size, potentially reducing statistical power for subtle effects. Thus, the cohort size (*N* = 18 IBD patients, with subgroups of *N* = 6–12 for specific analyses) was appropriate for proof-of-concept but limits the statistical power for subgroup comparisons by disease type, activity, or medication class. Conclusions regarding disease subtype differences and patient-specific response patterns are therefore exploratory and require confirmation in larger, prospectively stratified cohorts. The 24-hour culture window precludes analysis of chronic inflammatory or reparative processes, and mechanical stress from slice preparation induces NFκB activation that may confound baseline signaling though it reflects a biologically relevant response. Endoscopic biopsies yield limited tissue, and patient heterogeneity in medication use and disease location introduces variability. Control tissue, derived from macroscopically normal mucosa of colorectal cancer resections, served as non-inflamed control tissue, however, peri-tumoral microenvironmental effects on mucosal immune populations cannot be fully excluded, and comparisons with IBD tissue should be interpreted accordingly. Moreover, the ex vivo environment lacks systemic cues (hormonal, neural, vascular), and viability declines modestly over 24 h.

Future work will adapt the ePDTC platform to expanded pharmacological testing of both conventional and complementary therapies. The model’s accessibility makes it particularly suitable for patient-stratified pharmacology to correlate ex vivo drug responses with clinical outcomes, supporting precision medicine approaches as well as longitudinal studies tracking individual patients’ treatment responses over time. Incorporating additional immune cell populations and integration with innovative analytical methods, e.g., spatial transcriptomics or proteomics may yield deeper, cell-type-specific insights into drug responses.

## Supplementary Information

Below is the link to the electronic supplementary material.

Supplementary Material 1

## Data Availability

The data that support the findings of this study are available on request from the corresponding author.

## Declarations

### Conference presentation

Parts of this work were presented as an oral presentation at the Annual Congress of the German Society for Gastroenterology, Digestive and Metabolic Diseases (DGVS) - Viszeralmedizin 2025, Leipzig, Germany, September 2025.
