# Supplementary material for: Patient-derived tissue slice cultures from endoscopic biopsies as a translational ex vivo model for inflammatory bowel diseases
Source: Clin Exp Med. 2026 Jun 22;26(1):239. doi: 10.1007/s10238-026-02209-0 (PMC13287154; doi:10.1007/s10238-026-02209-0)
Supplement: Supplementary file 1 — Supplementary Material 1 [file 10238_2026_2209_MOESM1_ESM.docx]

**Supplementary Information**

**Online Resource 1: Model selection - Linear Mixed Model (LMM)**

The collected data follow a hierarchical structure (patient > biopsy > slice). To account for this structure, linear mixed models were calculated, as these models take into account both fixed effects (type/time of treatment) and random effects (patient and biopsy). Considering grouping structures in this way prevents biased standard errors and false positive results that can arise when dependencies are ignored^[[1]](#footnote-1)^. This structure is necessary in order to correctly model the non-independence of measurements within the same individual and the same biopsy, which traditional models such as ANOVA or linear regression are unable to perform. This mixed approach also allows full use of all data without loss of information, while taking the cluster structure into account.

The model is calculated in R using the following equation:

*model <- lmer(marker* ~ treat or time + (1|Patient/Biopsy), data = df)*

*e.g. antibody (CD68, ELA2, …) or inflammatory mediator

**Online Resource 2 - Medication of IBD patients at time of biopsy collection.**

Medication status at time of endoscopic biopsy. s.c. = subcutaneous administration; d = day; yr. = year; supp = suppository. 'Unknown' indicates patients for whom medication details could not be confirmed at time of biopsy. Percentages indicate proportion of patients receiving each medication class.

| **No.** | **Biologicals** | **Immuno-suppressants** | **Steroids** | **Mesalazine** | **Probiotics** | **unknown** |
| --- | --- | --- | --- | --- | --- | --- |
| **1** |  |  | Budesonide | x |  |  |
| **2** |  |  |  |  |  | x |
| **3** |  |  |  | x |  |  |
| **4** |  |  |  |  | Lactobacillus gasseri, Bifidobacterium longum |  |
| **5** |  | (Azathioprine, until 1 yr. prior to study inclusion) |  | x |  |  |
| **6** |  | Azathioprine |  |  |  |  |
| **7** | Vedolizumab (s.c./14d) |  | Prednisolone (10 mg),  Budesonide (3x 3 mg) |  |  |  |
| **8** | Adalimumab (s.c. 40 mg/14d) |  | Budesonid  (3 mg) |  |  |  |
| **9** |  |  |  |  | E. coli nissle |  |
| **10** |  | Azathioprine | Budesonide, Prednisolon (5 mg) |  |  |  |
| **11** |  |  |  | x (supp) |  |  |
| **12** |  |  |  |  |  | x |
| **13** |  | Ozanimod | Budesonide |  |  |  |
| **14** | Adalimumab (s.c./14d) |  |  |  |  |  |
| **15** |  |  |  | x (oral) |  |  |
| **16** |  | Ozanimod |  |  |  |  |
| **17** |  |  |  | x |  |  |
| **18** | Vedolizumab (s.c.) |  |  |  |  |  |
| ***%*** | *44.4%* | | *55.5%* | | *11.1%* | *11.1%* |

**Online Resource 3: Mediator release of untreated samples of N = 12 IBD patients after 24 h**

Data represent patient-level means after 24 h air-liquid interface culture. Mediator concentrations normalized to tissue slice area (pg/mL*µm²). Symbols indicate relative expression levels: + = low, ++ = moderate, +++ = high concentration relative to cohort median.

| **No.**  **Mediator** | **7** | **8** | **9** | **10** | **11** | **12** | **13** | **14** | **15** | **16** | **17** | **18** | **median** |
| --- | --- | --- | --- | --- | --- | --- | --- | --- | --- | --- | --- | --- | --- |
| **TNFα** | 5.74E-05 | 6.02E-05 | 2.54E-05 | 2.43E-05 | 8.22E-06 | 1.64E-05 | 9.92E-05 | 1.85E-05 | 3.76E-05 | 3.91E-04 | 5.49E-05 | 2.34E-05 | 3.15E-05 |
|  | ++ | ++ | + | + | + | + | +++ | + | ++ | +++ | ++ | + |  |
| **IL-33** | 1.69E-04 | 2.41E-05 | 1.49E-05 | 5.42E-05 | 1.03E-05 | 2.81E-05 | 1.48E-04 | 3.98E-05 | 1.25E-04 | 5.03E-04 | 1.80E-04 | 1.35E-04 | 8.96E-05 |
|  | ++ | + | + | + | + | + | ++ | + | ++ | +++ | ++ | ++ |  |
| **uPAR** | 5.98E-03 | 1.07E-03 | 3.66E-04 | 2.20E-03 | 2.12E-04 | 7.14E-05 | 5.71E-03 | 2.59E-03 | 2.65E-03 | 1.56E-02 | 7.40E-03 | 2.02E-03 | 2.39E-03 |
|  | +++ | + | + | ++ | + | + | +++ | ++ | ++ | +++ | +++ | + |  |
| **MIF** | 1.70E-01 | 3.75E-02 | 8.61E-02 | 6.59E-02 | 2.28E-02 | 2.35E-02 | 2.42E-01 | 4.18E-02 | 6.69E-02 | 6.07E-01 | 3.06E-01 | 9.49E-02 | 7.65E-02 |
|  | ++ | + | ++ | + | + | + | +++ | + | + | +++ | +++ | ++ |  |
| **IL-10** | 9.58E-05 | 5.34E-05 | 9.48E-06 | 2.89E-05 | 3.10E-06 | 2.47E-06 | 1.34E-05 | 6.37E-06 | 1.88E-05 | 3.02E-05 | 7.72E-06 | 6.26E-06 | 1.14E-05 |
|  | +++ | +++ | + | ++ | + | + | ++ | + | ++ | ++ | + | + |  |
| **MCP-1** | 7.41E-03 | 1.14E-03 | 2.17E-03 | 3.58E-03 | 2.47E-04 | 8.91E-04 | 1.12E-03 | 5.24E-04 | 2.87E-03 | 2.38E-03 | 1.41E-03 | 7.84E-04 | 1.27E-03 |
|  | +++ | + | ++ | +++ | + | + | + | + | +++ | ++ | ++ | + |  |
| **VEGF** | 1.40E-03 | 2.97E-04 | 9.46E-05 | 3.12E-04 | 2.72E-05 | 1.13E-05 | 3.46E-03 | 1.09E-03 | 1.38E-04 | 4.67E-03 | 2.71E-03 | 2.97E-04 | 3.04E-04 |
|  | ++ | ++ | + | ++ | + | + | +++ | ++ | + | +++ | +++ | ++ |  |
| **IL-1β** | 2.40E-04 | 1.91E-05 | 1.48E-05 | 3.17E-05 | 4.67E-06 | 7.58E-06 | 4.25E-05 | 2.60E-05 | 3.43E-05 | 1.67E-04 | 5.09E-05 | 2.70E-05 | 2.93E-05 |
|  | +++ | + | + | ++ | + | + | ++ | ++ | ++ | +++ | ++ | ++ |  |
| **IFNγ** | 1.56E-04 | 2.81E-05 | 1.71E-05 | 2.62E-05 | 4.38E-06 | 8.77E-06 | 1.56E-05 | 2.59E-05 | 1.41E-05 | 1.51E-04 | 1.10E-05 | 6.63E-06 | 1.64E-05 |
|  | +++ | ++ | ++ | ++ | + | + | ++ | ++ | ++ | +++ | + | + |  |
| **IL-4** | 1.81E-04 | 8.12E-05 | 1.00E-04 | 1.74E-04 | 6.64E-05 | 3.41E-05 | 2.84E-04 | 2.06E-04 | 2.94E-04 | 6.59E-04 | 4.90E-04 | 2.59E-04 | 1.93E-04 |
|  | ++ | + | + | ++ | + | + | ++ | ++ | ++ | +++ | +++ | ++ |  |
| **IL-17/IL-17A** | 1.12E-04 | 2.28E-05 | 9.62E-06 | 3.22E-05 | 4.62E-06 | 1.18E-05 | 1.13E-04 | 2.08E-05 | 2.93E-05 | 2.05E-04 | 2.12E-04 | 4.26E-05 | 3.08E-05 |
|  | +++ | + | + | ++ | + | + | +++ | + | ++ | +++ | +++ | ++ |  |
| **IL-2** | 4.51E-04 | 6.10E-05 | 3.73E-04 | 6.71E-05 | 3.07E-04 | 1.49E-04 | 1.22E-04 | 3.90E-05 | 4.97E-05 | 8.61E-05 | 6.61E-05 | 5.45E-05 | 7.66E-05 |
|  | +++ | + | +++ | + | +++ | +++ | ++ | + | + | ++ | + | + |  |
| **GM-CSF** | 1.27E-03 | 2.86E-05 | 3.02E-05 | 9.53E-05 | 2.10E-05 | 4.28E-05 | 8.09E-05 | 4.10E-05 | 8.50E-05 | 3.91E-04 | 1.91E-04 | 6.90E-05 | 7.49E-05 |
|  | +++ | + | + | ++ | + | + | ++ | + | ++ | +++ | +++ | ++ |  |
| **CXCL5/ENA-78** | 3.10E-02 | 2.04E-03 | 5.59E-04 | 3.40E-03 | 5.92E-04 | 1.44E-03 | 1.73E-03 | 3.79E-03 | 8.19E-03 | 1.21E-02 | 1.47E-02 | 1.19E-03 | 2.72E-03 |
|  | + | + | + | ++ | + | + | + | ++ | +++ | +++ | +++ | + |  |
| **CXCL9/MIG** | 2.53E-03 | 9.61E-04 | 9.74E-04 | 1.72E-03 | 5.06E-04 | 3.18E-04 | 2.68E-03 | 1.58E-03 | 2.53E-03 | 6.20E-03 | 5.08E-03 | 2.76E-03 | 2.13E-03 |
|  | ++ | + | + | + | + | + | ++ | + | ++ | +++ | +++ | +++ |  |
| **Lipocalin-2** | 9.04E-02 | 1.75E-02 | 1.35E-02 | 2.07E-02 | 3.92E-03 | 6.49E-03 | 6.09E-02 | 1.19E-02 | 1.39E-02 | 1.33E-01 | 2.22E-01 | 1.13E-02 | 1.57E-02 |
|  | +++ | ++ | + | ++ | + | + | +++ | + | + | +++ | +++ | + |  |
| **G-CSF** | 3.38E-02 | 3.27E-03 | 5.10E-05 | 5.14E-03 | 4.21E-04 | 1.24E-03 | 4.18E-03 | 1.83E-03 | 3.45E-02 | 3.73E-02 | 4.64E-02 | 1.42E-03 | 3.72E-03 |
|  | +++ | + | + | ++ | + | + | ++ | + | +++ | +++ | +++ | + |  |
| **TFF3** | 2.43E-02 | 4.00E-02 | 1.20E-02 | 7.23E-02 | 1.90E-02 | 4.47E-03 | 5.90E-02 | 4.61E-02 | 3.62E-02 | 1.18E-01 | 1.52E-01 | 1.80E-02 | 3.81E-02 |
|  | + | ++ | + | +++ | + | + | ++ | ++ | ++ | +++ | +++ | + |  |
| **GDF-15** | 1.25E-03 | 6.96E-04 | 1.92E-04 | 1.44E-03 | 8.14E-04 | 1.09E-04 | 9.05E-03 | 8.80E-04 | 1.02E-03 | 7.30E-03 | 2.83E-03 | 9.87E-04 | 1.00E-03 |
|  | ++ | + | + | ++ | + | + | +++ | ++ | ++ | +++ | ++ | ++ |  |
| **Angiogenin** | 8.89E-03 | 3.28E-03 | 1.82E-03 | 6.52E-03 | 9.97E-04 | 4.10E-04 | 1.17E-02 | 2.95E-03 | 4.28E-03 | 2.20E-02 | 1.01E-02 | 3.68E-03 | 3.98E-03 |
|  | +++ | + | + | +++ | + | + | +++ | + | ++ | +++ | +++ | ++ |  |
| **IL-23** | 3.11E-03 | 8.92E-04 | 5.67E-04 | 1.27E-03 | 3.52E-04 | 1.45E-04 | 5.32E-03 | 1.57E-03 | 2.42E-03 | 1.05E-02 | 7.38E-03 | 2.93E-03 | 2.00E-03 |
|  | ++ | + | + | + | + | + | +++ | + | ++ | +++ | +++ | ++ |  |
| **IL-6** | 1.50E-02 | 1.24E-03 | 1.40E-04 | 2.98E-03 | 1.61E-04 | 2.00E-03 | 1.01E-03 | 1.15E-04 | 2.17E-03 | 3.52E-03 | 1.15E-03 | 3.29E-05 | 1.20E-03 |
|  | +++ | ++ | + | +++ | + | +++ | ++ | + | +++ | +++ | ++ | + |  |
| **IL-8** | 1.37E-01 | 3.59E-02 | 3.05E-02 | 2.89E-01 | 1.59E-02 |  | 3.37E-02 | 1.44E-02 | 1.08E-01 | 1.32E-01 | 6.30E-02 | 4.99E-03 | 3.59E-02 |
|  | +++ | ++ | ++ | +++ | + |  | ++ | + | +++ | +++ | ++ | + |  |
| **Serpin-E1** | 1.06E-02 | 2.70E-03 | 1.04E-03 | 1.67E-03 | 3.55E-04 | 4.30E-03 | 3.93E-03 | 4.63E-04 | 2.51E-03 | 9.02E-03 | 2.28E-03 | 3.29E-04 | 2.39E-03 |
|  | +++ | ++ | + | ++ | + | +++ | +++ | + | ++ | +++ | ++ | + |  |

1. Da Silveira LTY, Ferreira JC, Patino CM. Mixed-effects model: a useful statistical tool for longitudinal and cluster studies. J Bras Pneumol. 2023;49(2):e20230137. doi:10.36416/1806-3756/e20230137 [↑](#footnote-ref-1)
